# Endothelial-derived small extracellular vesicles support B-cell acute lymphoblastic leukemia development

**DOI:** 10.1007/s13402-023-00855-0

**Published:** 2023-09-26

**Authors:** Dan Huang, Yamin Yuan, Liyuan Cao, Difan Zhang, Yu Jiang, Yaping Zhang, Chiqi Chen, Zhuo Yu, Li Xie, Yujuan Wei, Jiangbo Wan, Junke Zheng

**Affiliations:** 1 Hongqiao International Institute of Medicine, Shanghai Tongren Hospital, Key Laboratory of Cell Differentiation and Apoptosis of Chinese Ministry of Education, Faculty of Basic Medicine, Shanghai Jiao Tong University School of Medicine, Shanghai, 200025 China; 2 Department of Hematology, Xinhua Hospital, Affiliated to Shanghai, Jiao Tong University School of Medicine, Shanghai, 200092 China

**Keywords:** VPS33B, B-ALL development, SEVs, ANGPTL2, Endothelial cells

## Abstract

**Purpose:**

The bone marrow niche plays an important role in leukemia development. However, the contributions of different niche components to leukemia development and their underlying mechanisms remain largely unclear.

**Method:**

Cre/LoxP-based conditional knockout technology was used to delete VPS33B or ANGPTL2 gene in niche cells. Murine B-ALL model was established by overexpressing the N-Myc oncogene in hematopoietic stem progenitor cells. The frequency of leukemia cells and immunophenotypic B220^+^ CD43^+^ LICs was detected by flow cytometry. SEVs was isolated by sequential centrifugation and mass spectrometry was performed to analyze the different components of SEVs. Immunoprecipitation and western blot were used to measure the interaction of VPS33B and ANGPTL2.

**Results:**

Here, we showed that specific knockout of vascular protein sorting 33b (Vps33b) in endothelial cells (ECs), but not megakaryocytes or mesenchymal stem cells, resulted in a significant decrease in the secretion of small extracellular vesicles (SEVs) and a delay in the development of B-cell lymphoblastic leukemia (B-ALL). Vps33b knockdown endothelial cells contained much lower levels of SEVs that contained angiopoietin-like protein 2 (ANGPTL2) than the control cells. Importantly, conditional knockout of Angptl2 in ECs significantly delayed B-ALL progression. Moreover, C-terminal region of ANGPTL2 (aa247-471) could directly interact with Sec1-like domain 1 of VPS33B (aa1-aa146). We further demonstrated that the point mutations R399H and G402S in ANGPTL2 led to a dramatic decrease in the secretion of ANGPTL2-SEVs. We also showed that wild-type ANGPTL2-containing SEVs, but not mutant ANGPTL2-containing SEVs, significantly enhanced B-ALL development.

**Conclusion:**

In summary, our findings indicate that the secretion of ANGPTL2-containing SEVs in ECs sustains the leukemogenic activities of B-ALL cells, which is fine-tuned by the direct interaction of VPS33B and ANGPTL2. These findings reveal that niche-specific SEVs play an important role in B-ALL development.

**Graphical abstract:**

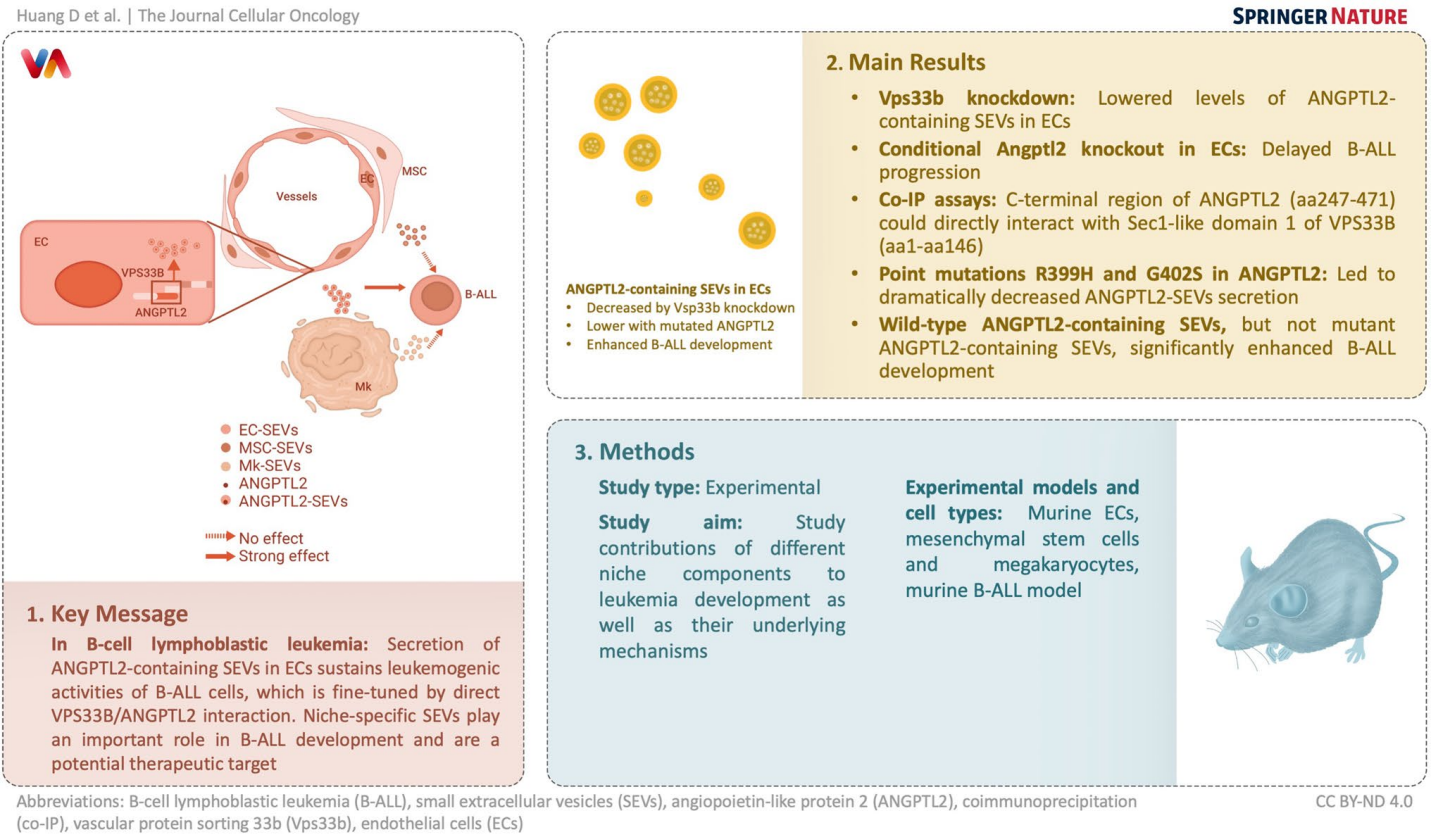

**Supplementary Information:**

The online version contains supplementary material available at 10.1007/s13402-023-00855-0.

## Introduction

B-cell acute lymphoblastic leukemia (B-ALL) is a type of malignant hematopoietic disorder that originates from hematopoietic stem progenitor cells and commonly occurs in youth and adults. Chemotherapy is routinely used for B-ALL treatment and usually achieves an overall response rate of approximately 70–80%. However, approximately 20% of B-ALL patients still relapse due to resistance to chemotherapeutic drugs (such as cytarabine and anthracycline) [1–3]. Many studies have suggested that chemoresistance and relapse of B-ALL may be closely related to leukemia initiating cells (LICs). It has been reported that immunophenotypic CD34^+^CD19^+^ LICs may exist in human primary B-ALL bulk cells, which are responsible for the initiation, progression, relapse and drug resistance of B-ALL [4].

Emerging evidence has shown that various components in the bone marrow (BM) niche may play a key role in the development of leukemia [5, 6]. The BM niche contains both different cell types (such as osteoblasts, mesenchymal stem cells, endothelial cells, adipocytes and megakaryocytes) and soluble niche factors (such as growth factors, cytokines, hormones, and metabolites) essential for the maintenance of LICs activities [7–9]. Several studies have also suggested that leukemia cells reside in a unique BM niche, which is rebuilt to escape chemotherapy [5, 6, 10]. However, it also remains unclear which niche components are required for LICs activities and how these niche factors are processed and released to support leukemogenesis.

Our previous studies showed that small extracellular vesicles (SEVs) secreted by leukemia cells could rebuild the BM niche in an autocrine or paracrine manner [11]. SEVs have multiple physiological and pathological effects on recipient cells through the delivery of different cargo types, including mRNA, microRNA (miRNA) and proteins [12]. For example, SEVs secreted by B-ALL cells altered the tumor microenvironment by inducing a metabolic switch in BM stromal cells [13]. Other studies have also shown that SEVs secreted by AML cells inhibit normal hematopoietic and bone formation by upregulating DKK1 expression in bone marrow stromal cells [14]. The SEVs secreted by leukemia cells could also enhance their proliferation in an autocrine manner [11, 15, 16]. Corrado et al. observed that SEVs from chronic myeloid leukemia cells could stimulate the expression of IL-8 in BM stromal cells, which further promoted the development of chronic myeloid leukemia through its receptors CXCR1 and CXCR2 [15].

Although numerous studies have demonstrated the effects of SEVs derived from leukemia cells on the bone marrow microenvironment, there is still limited understanding of how SEVs released by a specific niche cell type regulate leukemia cells [11, 14, 17, 18]. Interestingly, our previous studies have demonstrated that vacuolar protein sorting 33b (VPS33B) plays a vital role in the maturation and secretion of SEVs or exosomes to support AML development [19]. VPS33B belongs to the Sec1/Munc18 family of class C vacuolar protein sorting proteins, which is indispensable for vesicle-mediated protein trafficking to lysosomal compartments and membrane docking/fusion reactions between multivesicular bodies (MVBs) and the plasma membrane [20]. VPS33B mutations lead to severe defects in humans, including arthrogryposis, renal dysfunction and cholestasis, termed ARC syndrome in the clinic [21]. It has also been reported that VPS33B mutations may be tightly involved in tumorigenesis [22, 23]. Because different types of leukemia are characterized by diverse genetic and epigenetic alterations and distinct cell origins [19, 24, 25], SEVs from a specific niche cell type may also play differential roles in the development of different leukemias. Herein, we used several cell-type-specific murine Cre lines to conditionally knockout Vps33b in endothelial cells (ECs), BM mesenchymal stem cells (MSCs) and megakaryocytes (MKs) to determine the roles of different niche cell-derived exosomes in B-ALL development.

## Results

### VPS33B-mediated SEV secretion from endothelial cells enhances B-ALL progression

**Fig. 1:**
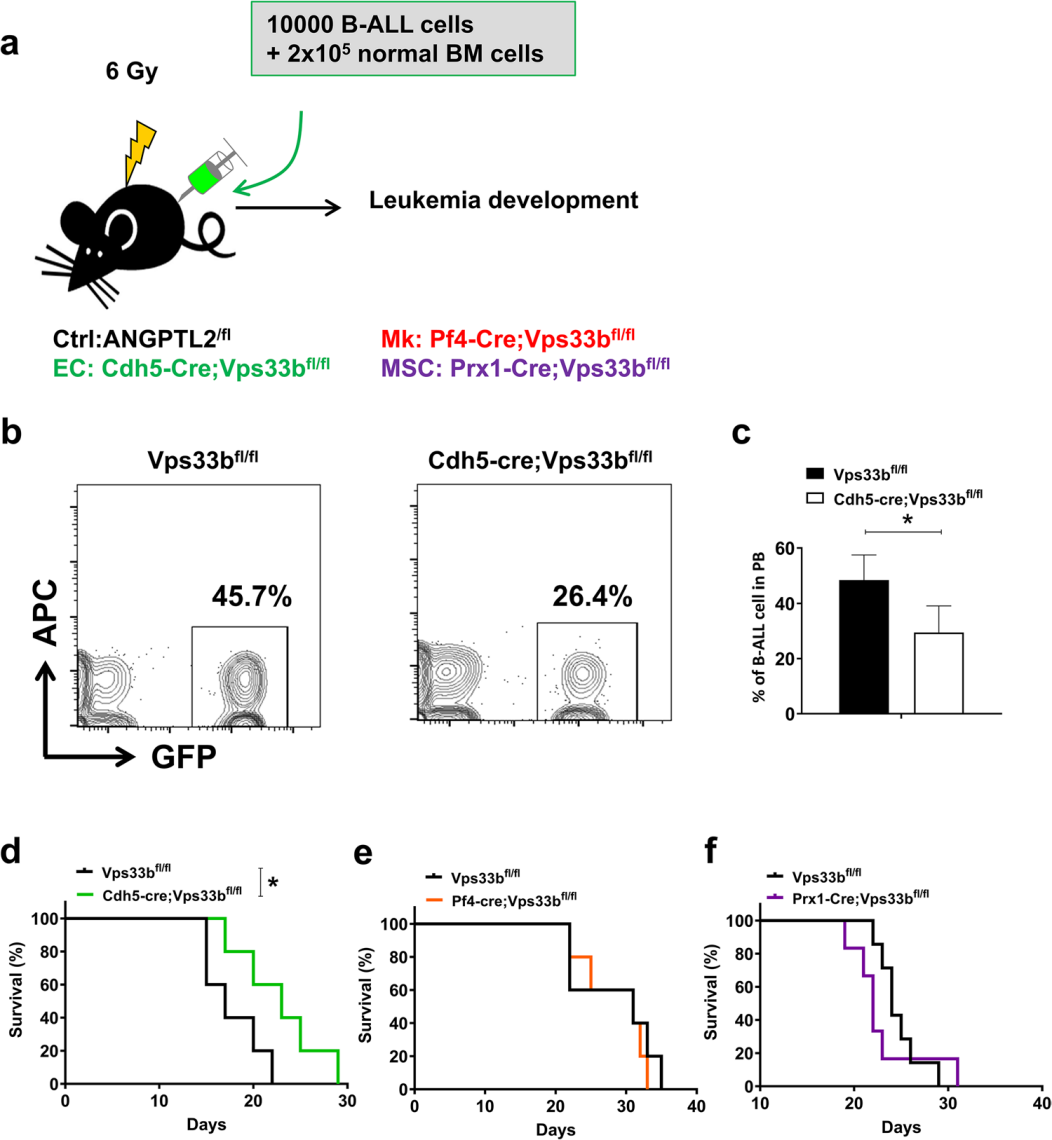
Reduced endothelial cell-derived exosome secretion prolongs survival in B-ALL mice. (**a**) The experimental design for the B-ALL murine model with Vps33b deletion in niche cells, including endothelial cells (Cdh5-Cre;Vps33b^fl/fl^), megakaryocytes (Pf4-Cre;Vps33b^fl/fl^), and mesenchymal stem cells (Prx1-Cre;Vps33b^fl/fl^). (b-c) Representative plots for flow cytometric analysis of the percentage of GFP^+^ leukemia cells in the peripheral blood (PB) of recipients 20 days after transplantation (**b**). Quantification data in Panel b are shown (**c**; *n* = 5; *P < 0.05, Student’s t test). (d-f) Overall survival in B-ALL recipient mice with Vps33b deletion in niche cells, including endothelial cells (Cdh5-Cre;Vps33b^fl/fl^, **d**), megakaryocytes (Pf4-Cre;Vps33b^fl/fl^, **e**), and mesenchymal stem cells (Prx1-Cre;Vps33b^fl/fl^, **f**). (*n* = 5–7; *P < 0.05, log-rank test)

To determine which niche cell components produce specific SEVs to promote B-ALL development, we generated several tissue-specific Vps33b knockout mice by cross-breeding with Vps33b^fl/fl^ mice with tissue-specific Cre transgenic mice, including Cdh5-Cre (endothelia specific [26]), Prx1-Cre (mesenchymal stem cell specific [27]) and Pf4-Cre (megakaryocyte specific [28]) mice, which can decrease SEV secretion from related niche cells. We then established an N-Myc-induced murine B-ALL model, as evidenced by the expression of the B220 B-cell marker but not T-cell and myeloid cell markers (Fig. S1a-b). We further injected 10,000 B-ALL cells together with 2 × 10^5^ total BM cells into lethally irradiated recipients, including Cdh5-Cre;Vps33b^fl/fl^, Prx1-Cre;Vps33b^fl/fl^ and Pf4-Cre;Vps33b^fl/fl^ mice, and monitored the effects on leukemia development (Fig. 1a). Vps33b^fl/fl^ mice were used as the control recipients (Ctrl) in these experimental settings. Interestingly, B-ALL development was significantly delayed, as evidenced by the reduced GFP^+^ leukemia cells in the peripheral blood (45.7% vs. 26.4%, respectively; Fig. 1b-c). More importantly, we found that the overall survival of Cdh5-Cre;Vps33b^fl/fl^ recipients was markedly extended (22 days for Vps33b^fl/fl^ and 29 days for Cdh5-Cre;Vps33b^fl/fl^ recipients; Fig. 1d). However, there was no effect on B-ALL development after Vps33b was specifically deleted in MSCs and MKs, as revealed by no changes in B-ALL cell frequency in the peripheral blood or overall survival in the recipient (Fig. 1e-1f and Fig. S1c-f).

### ANGPTL2-containing SEVs maintain the leukemogenic activities of murine B-ALL cells

**Fig. 2:**
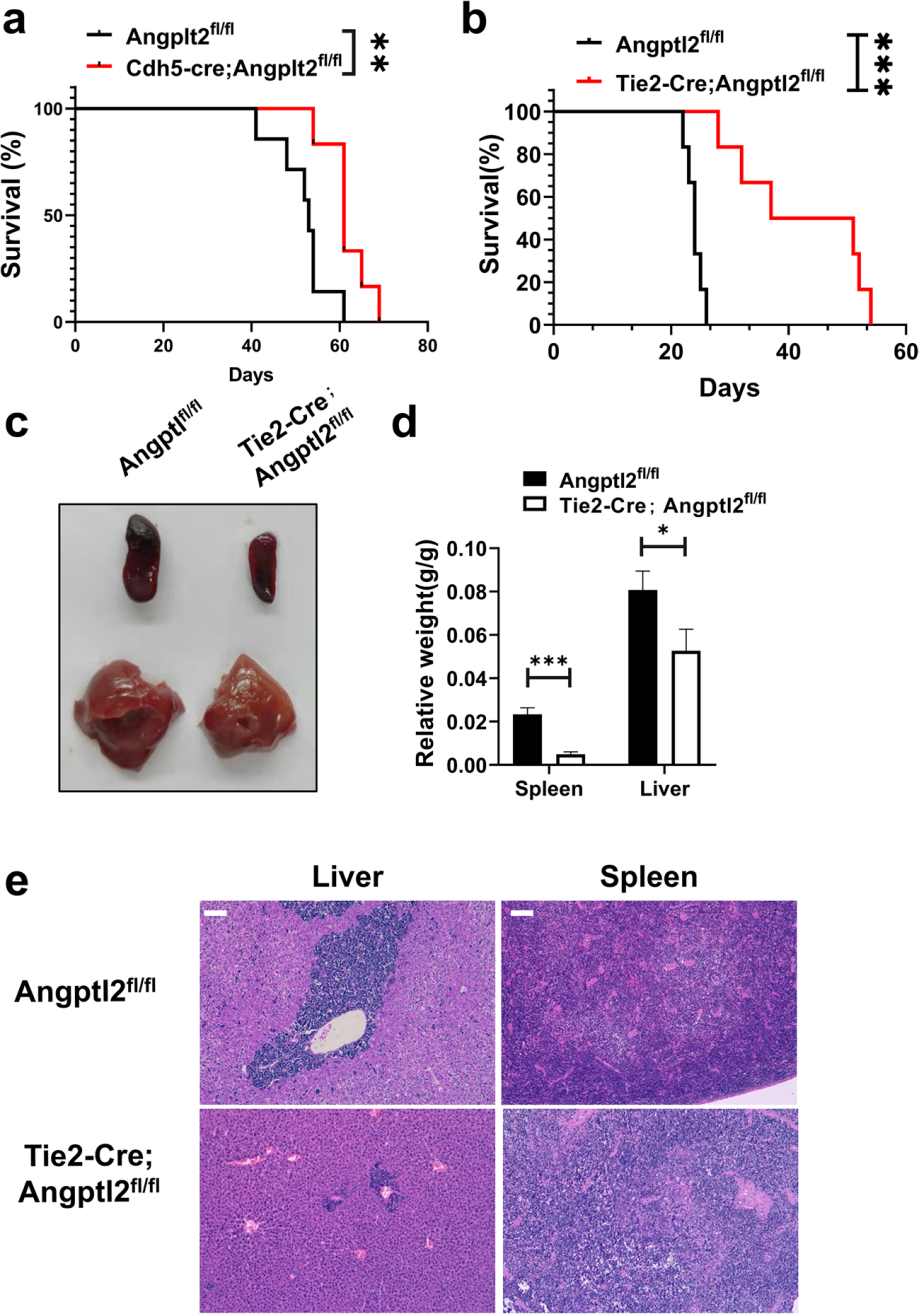
ANGPTL2-containing SEVs maintain the leukemogenic activities of murine B-ALL cells. (**a**) B-ALL cells were injected into Cdh5-Cre;Angptl2^fl/fl^ recipient mice and Vps33b^fl/fl^ control mice, and their overall survival was analyzed. (**b**) B-ALL cells were injected into Tie2-Cre;Angptl2^fl/fl^ recipient mice and Vps33b^fl/fl^ control mice, and their overall survival was analyzed. (c-d) Representative images of spleens and livers of recipients (**c**) and relative weights (**d**) are shown 20 days after B-ALL cell transplantation. (*n* = 3–7; *P < 0.05, ***P < 0.001, Student’s t test). (**e**) Histological hematoxylin/eosin staining of the livers and spleens of recipients in Panel c. Scale bar, 20 µm

To further determine which components of SEVs play important roles in B-ALL development, we performed mass spectrometry analysis of SEVs from Vps33b knockdown murine endothelial cells as described in our previous study [19]. We found that the pathway of extracellular exosomes was significantly changed, and one of the components, ANGPTL2, was highly enriched in the SEVs (Fig. S2a-b). To test whether ANGPTL2-containing SEVs from ECs enhanced B-ALL progression, we generated Cdh5-Cre;Angptl2^fl/fl^ mice to specifically delete Angptl2 in ECs and transplanted B-ALL cells into these recipient mice. Interestingly, we found that overall survival was markedly extended in the Cdh5-Cre;Angptl2^fl/fl^ recipients compared with the control mice (61 days for Cdh5-Cre; Angptl2^fl/fl^ recipients and 53 days for Angptl2^fl/fl^, Fig. 2a). Consistently, overall survival was markedly extended in the Tie2-Cre;Angptl2^fl/fl^ recipients (another murine model with Angptl2 deletion in ECs) compared with the control mice (44 days for Tie2-Cre; Angptl2^fl/fl^ recipients and 24 days for Angptl2^fl/fl^, Fig. 2b). Reduced infiltration was also observed in the Tie2-Cre;Angptl2^fl/fl^ recipients transplanted with B-ALL cells, as evidenced by the decreased relative weights of their spleens and livers (Fig. 2c-d) and the less infiltrated leukemia (Fig. 2e). These results indicate that ANGPLT2-containing SEVs secreted from endothelial cells support B-ALL development.

### The C-terminal region of ANGPTL2 directly interacts with VPS33B

**Fig. 3:**
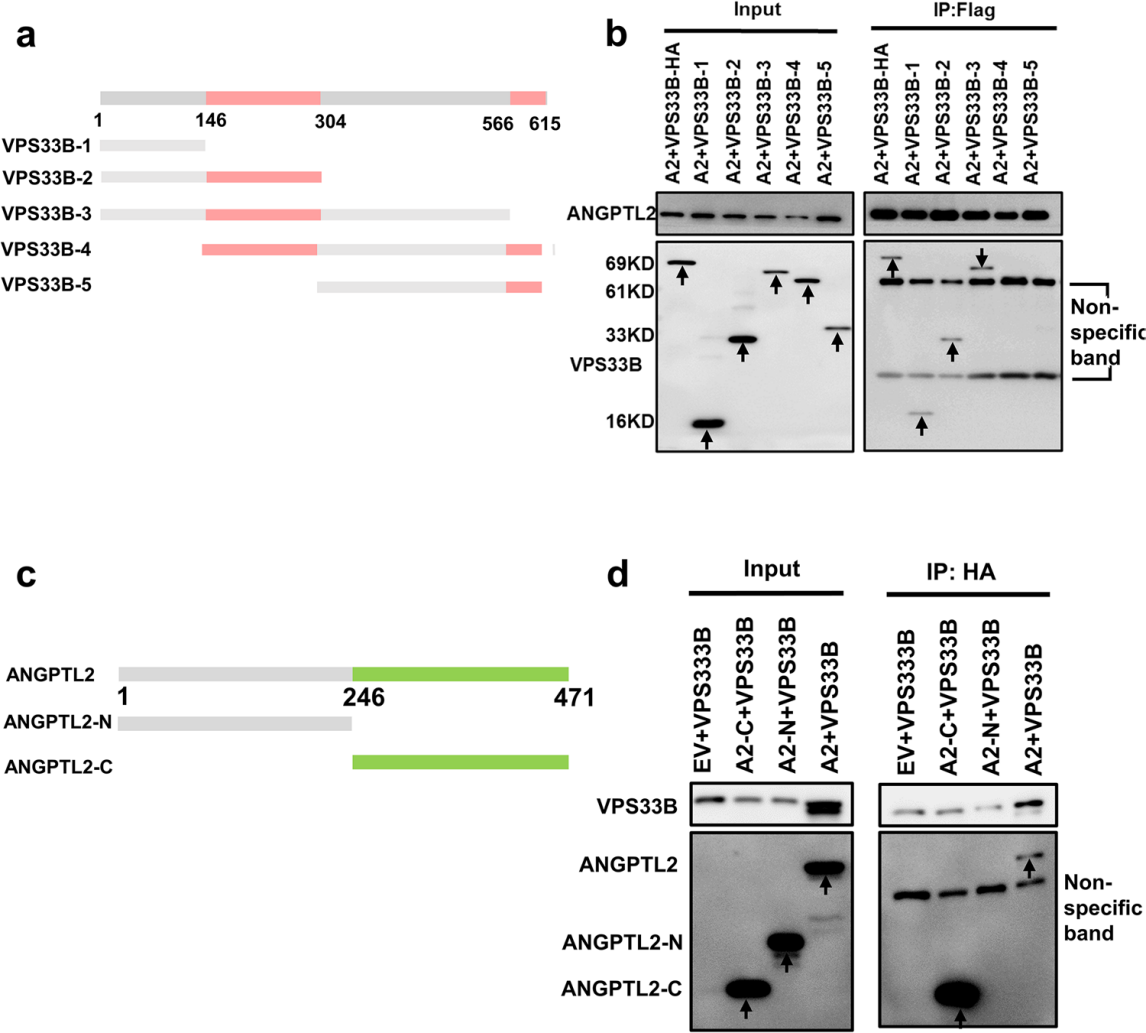
The C-terminal region of ANGPTL2 directly interacts with VPS33B. (**a**) Diagram for the truncated forms of VPS33B: aa1–146 (16 kDa), aa1-304 (33 kDa), aa1-566 (62 kDa), aa146-615 (59 kDa) and aa304-617. (**b**) IP experiments were performed to examine the interaction of HA-tagged mutant VPS33B with Flag-ANGPTL2 in HEK293T cells. (**c**) Diagram of the truncated forms of ANGPTL2: ANGPTL2-N-terminal (Flag-ANGPTL2-N) and ANGPTL2-C-terminal (Flag-ANGPTL2-C). (**d**) IP experiments were performed to examine the interaction of HA-tagged VPS33B with Flag-tagged mutant ANGPTL2 in HEK293T cells

Our previous studies have shown that VPS33B can regulate the release of ANGPTL2-containing SEVs [19]. We then attempted to assess which domains of VPS33B and ANGPTL2 are critical for their interaction. Several truncations of VPS33B were first generated to decipher which region of VPS33B contributed to the interplay with ANGPTL2 (Fig. 3a). We then transfected full-length VPS33B plasmid (HA-VPS33B-WT) or five VPS33B truncated plasmids (HA-VPS33B-1, HA-VPS33B-2, HA-VPS33B-3, HA-VPS33B-4, HA-VPS33B-5) and Flag-ANGPTL2 plasmid into HEK293T cells and performed co-IP experiments. Interestingly, we found that only the truncations containing the first 146 amino acids (aa 1-aa 146) of VPS33B (VPS33B-1, VPS33B-2 and VPS33B-3) could directly interact with ANGPTL2 (Fig. 3b). However, other truncations (VPS33B-4 and VPS33B-5) containing Sec1-like domains did not interact with ANGPTL2. Next, we sought to determine the potential region of ANGPTL2 that could interact with VPS33B. We generated both the N-terminal region (aa1-aa246) and the C-terminal region (aa247-aa471) of ANGPTL2 (Fig. 3c) and performed co-IP experiments and showed that only the C-terminal region of ANGPTL2 directly interacted with VPS33B (Fig. 3d). Taken together, these data revealed that the ANGPTL2 and VPS33B interaction occurs at the C-terminal region of ANGPTL2 (aa 247–471) and the first 146 amino acids of VPS33B.

### VPS33B-ANGPTL2 interaction is required for the secretion of ANGPTL2-containing SEVs

**Fig. 4:**
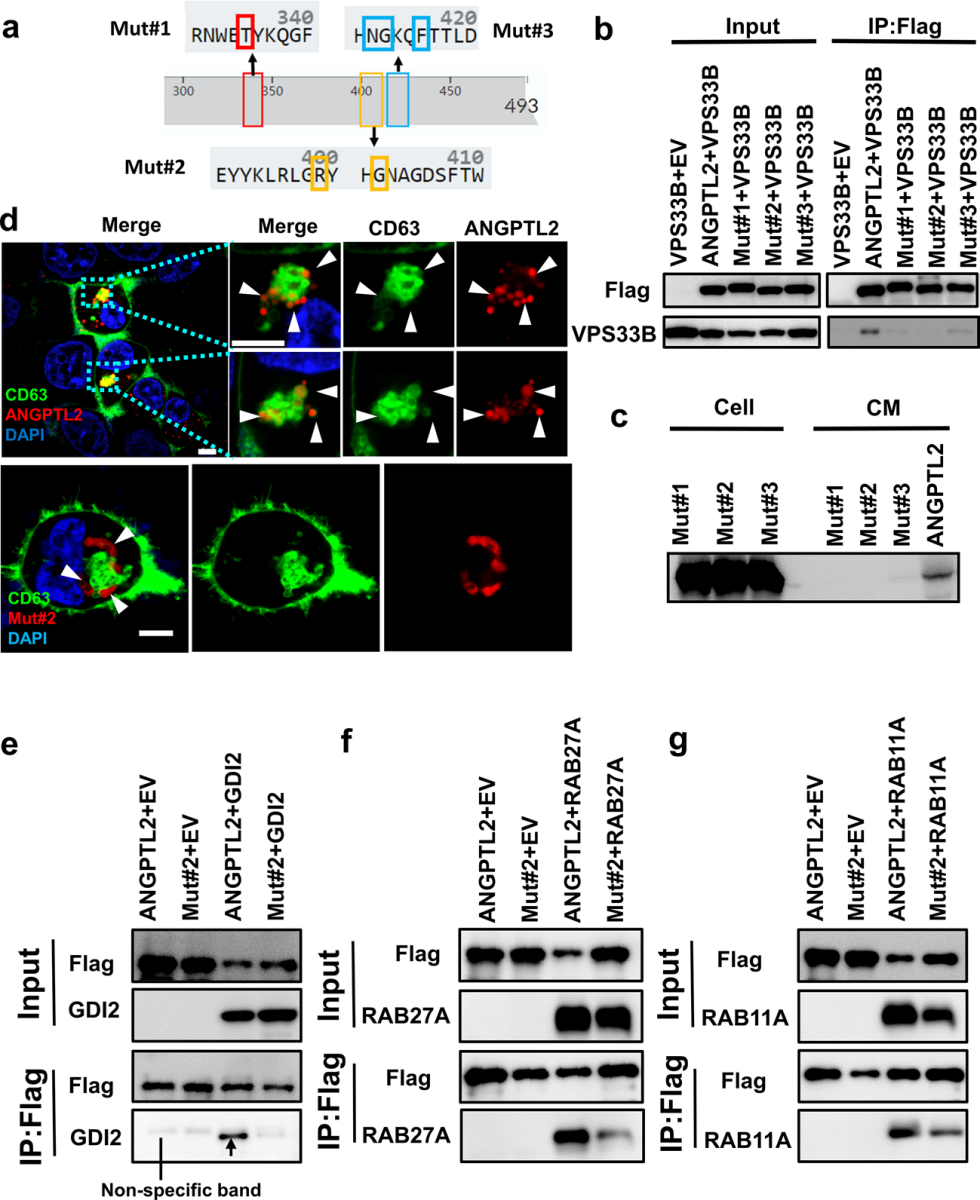
VPS33B-ANGPTL2 interaction is required for the secretion of ANGPTL2-containing SEVs. (**a**) Diagram of the mutations in the ANGPTL2 C-terminal domain. Mut#1 has the mutation at the T335 residue. Mut#2 has mutations at the R399 and G402 residues. Mut#3 has mutations at the N412, G413 and F416 residues. (**b**) An immunoprecipitation assay was performed to examine the interaction of the FLAG-tagged ANGPTL2 mutant with HA-VPS33B in HEK293T cells. (**c**) Representative images of the colocalization of CD63 with ANGPTL2 or ANGPTL2 mutants in HEK293T cells. (**d**) First, HEK293T cells were transfected with WT ANGPTL2 and its mutants, followed by the examination of ANGPTL2 protein levels in HEK293T cells and conditioned medium (CM) by Western blot. (e–g) Interaction of WT ANGPTL2 and ANGPTL2 mutants with GDI2 (**e**), RAB27A (**f**) and RAB11A (**g**) was evaluated by immunoprecipitation

To further identify the specific interaction site between ANGPTL2 and VPS33B, we examined their potential binding capacities with different ANGPTL2 mutations at the C-terminal region (aa247-aa471) that occur frequently in human cancers, as revealed by public data settings (such as the Catalogue of Somatic Mutations in Cancer, COSMIC). Interestingly, we identified three ANGPTL2 mutants (ANGPTL2-Mut#1-#3) with reduced binding abilities to VPS33B, especially ANGPTL2-Mut#2, as determined by co-IP assays (Fig. 4a-b). To explore the function of the VPS33B-ANGPTL2 interaction in the maturation and release of ANGPTL2-containing SEVs, we overexpressed WT ANGPTL2 and its mutants (ANGPTL2-Mut#1-#3) in HEK293T cells and demonstrated that ANGPTL2 expression in the supernatant was markedly decreased (Fig. 4c). In addition, confocal microscopy showed that ANGPTL2-Mut#2 protein was not colocalized with CD63, a marker of SEVs (Fig. 4d), indicating that the VPS33B-ANGPTL2 interaction is critical for the secretion of ANGPTL2-containing SEVs.

We previously showed that VPS33B can directly interact with the GDI2/RAB11A/RAB27A pathway to regulate SEV secretion [11]. We then sought to examine whether VPS33B also regulates the maturation and release of ANGPTL2-SEVs via the GDI2/RAB11A/RAB27A pathways. Interestingly, ANGPTL2-Mut#2 had notably reduced binding capacities to RAB27A and GDI2 but not to RAB11A, as revealed by the co-IP experiment. These data indicate that RAB27A and GDI2, but not RAB11A, are important for the secretion of ANGPTL2-containing SEVs (Fig. 4e-g).

### ANGPTL2-containing SEVs support B-ALL development

**Fig. 5:**
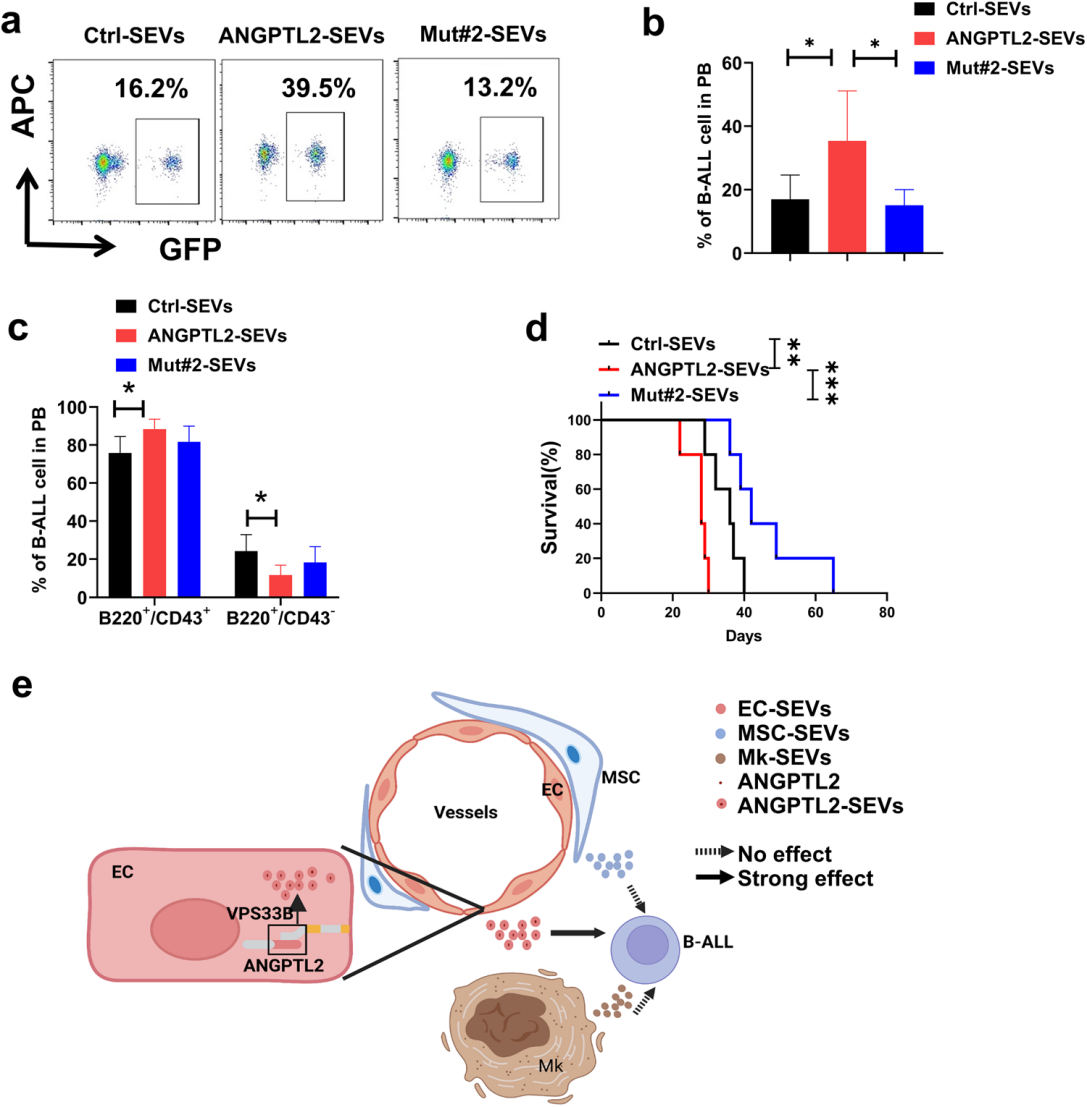
ANGPTL2-containing SEVs support B-ALL development. (**a**-**b**) Representative plots for the flow cytometric analysis (A) of the percentage of GFP^+^ leukemia cells in the peripheral blood (PB) of recipients 20 days after transplantation. Quantification data are shown in Panel B (B; *n* = 5–6; *P < 0.05, one-way ANOVA with Tukey’s multiple-comparison test). (**c**) Percentage of B220 and CD43 cells in the PB of recipients 20 days after transplantation. (*n* = 6; *P < 0.05, two-way ANOVA with Sidak’s multiple comparison test). (**d**) The overall survival of recipients receiving B-ALL cells cocultured with ctrl-SEVs, ANGPTL2-SEVs and ANGPTL2-mutant-SEVs is shown (*n* = 6; **P < 0.01, ***P < 0.001, log-rank test). (**e**) The working model related to how EC-derived SEVs enhance B-ALL development

To further examine the biological function of VPS33B-mediated ANGPTL2-containing SEVs in leukemogenesis, we cultured B-ALL cells with purified ANGPTL2-containing SEVs, ANGPTL2-mut#2-containing SEVs and Ctrl SEVs, followed by transplantation into recipient mice. Interestingly, ANGPTL2-containing SEVs significantly enhanced B-ALL development, as displayed by much higher leukemia cell frequencies in peripheral blood (Fig. 5a-b), much higher frequencies of immunophenotypic B220^+^CD43^+^ LICs (Fig. 5c) and reduced overall survival (Fig. 5d) compared to Ctrl SEVs. However, ANGPTL2-mut#2-containing SEVs had no effects on B-ALL progression (Fig. 5a-d), which further indicated that ANGPTL2 was mainly secreted as SEVs in the BM niche to maintain leukemogenic activities. In summary, we demonstrate that endothelial cell-derived ANGPTL2-containing SEVs support B-ALL development, which is fine-tuned by VPS33B. VPS33B directly interacts with the C-terminus of ANGPTL2 to regulate the secretion of SEVs (Fig. 5e).

## Discussion

How niche cell-derived SEVs regulate leukemogenesis remains largely unknown. Herein, we used several different niche cells type-specific murine Cre lines to conditionally delete *Vps33b* in endothelial cells, MSCs and MKs and demonstrated that only endothelial cells support B-ALL development. However, we also believe that other niche cells, including OPCs, osteoblast cells, sympathetic nerves, nonmyelinating Schwann cells, and adipocytes [6–8, 29, 30], may be involved in the secretion of SEVs to support B-ALL development. Although emerging evidence has demonstrated that the BM niche plays a vital role in B-ALL development, little is known about the function of SEVs secreted from different niche cells in leukemogenesis. Therefore, more efforts are required to elucidate the role of VPS33B in other niche cells in supporting B-ALL cell activities.

Several studies have shown that ECs play an important role in leukemogenesis. It has been reported that B-ALL cells may reside in a specialized vascular niche, and the specialized endothelium provides a unique microenvironment for the early metastasis of certain tumors in bone marrow [31]. We also showed that LIC-enriched B-ALL cells tended to reside in the vascular endothelial niche in the BM [31]. Consistent with these data, our study also provided several lines of evidence for the roles of EC-derived SEVs in B-ALL development, which showed that ANGPTL2-containing SEVs secreted by ECs were critical for leukemogenesis. However, BM ECs are heterogeneous and have different subtypes, including sinusoidal ECs, arterial ECs, and arteriolar/capillary ECs [32–34]. These three EC subtypes have different locations and secrete different levels of cytokines, which are likely involved in the maintenance of leukemogenic activities in the BM. Future studies should use specific Cre mice to confirm the potential effects of SEVs from different ECs on B-ALL progression and to unravel the underlying mechanisms by which EC-derived SEVs regulate different types of leukemia.

As a secretory protein, ANGPTL2 plays an important role in physiological and pathological situations, such as inflammation, lipid metabolism, vascular remodeling, and tumorigenesis [35–41]. In this study, we demonstrated that EC-derived SEVs contained high levels of ANGPTL2, which can further promote B-ALL development. In particular, our group recently showed that EC-derived ANGPLT2 is required for the self-renewal ability of HSCs [42], suggesting that ANGPTL2 has different effects on different cell types, such as HSCs or leukemia cells. More efforts are required to fully illustrate the underlying regulatory networks of ANGPTL2 in normal hematopoiesis and hematomalignancies to provide new insights for the treatment of leukemia. We also performed a screening of ANGPTL2 mutations in the COSMIC dataset and showed that the point mutations R399H and G402S (Mut#2) can specifically reduce the secretion of ANGPTL2-containing SEVs. In this study, we demonstrated that ANGPTL2-containing SEVs released from ECs support B-ALL development while ANGPTL2-mut#2-containing SEVs had no effects on B-ALL progression. However, no mutations of ANGPTL2 were found in B-ALL, as revealed by public data settings (such as COSMIC or cBioPortal). One explanation is that the mutation of ANGPTL2 may occur mainly in endothelial cells rather than B-ALL cells. In addition, our data implied that the mutation of ANGPTL2 decreases the release of ANGPTL2-SEVs, which leads to the reduced effect of ANGPTL2 on enhancing B-ALL development. Therefore, further efforts are required to unravel the roles of ANGPTL2 mutants in ECs in the initiation and progression of different cancer types, especially through the related release of ANGPLT2-SEVs. Moreover, The R399H ANGPTL2 mutation frequently occurs in endometrial cancer, while the G402S ANGPTL2 mutation often appears in colorectal cancer. It will be very important to unravel the potential functions of ANGPTL2 mutants in different types of cancers and whether these effects are dependent on the exosomal secretion of ANGPTL2.

In summary, we herein demonstrate that EC-derived SEVs play important roles in the development of murine B-ALL, which is tightly regulated by VPS33B. The first 146 AAs of VPS33B and the C-terminus of ANGPTL2 are critical for their direct interaction and are required for the release of ANGPTL2-containing SEVs from endothelial cells. Certain mutations of ANGPTL2 in many human cancers may result in a marked reduction in SEV secretion, which further contributes to tumorigenesis. These data suggest that targeting leukemia niche components, such as ANGPTL2-containing SEVs, may be a potential treatment for B-ALL or other cancer types.

## Methods and materials

### Study approval

Animal experiments were approved by our institutes and complied with the Guideline for Animal Care at the Shanghai Jiao Tong University School of Medicine.

### Mice

For specific deletion of Vps33b in murine ECs, MSCs and MKs, Vps33b^fl/fl^ mice were crossed with Cdh5-Cre, Pf4-Cre and Prx1-Cre mice. Cre-Vps33b^fl/fl^ littermate mice served as controls. For specific deletion of Angptl2 in murine ECs, Angptl2^fl/fl^ mice were crossed with Cdh5-Cre and Tie2-cre mice. Angptl2^fl/fl^ littermate mice served as controls. 6- to 8-week-old C57BL/6 mice were purchased from the Shanghai SLAC Laboratory Animal Co. Ltd. All mice used in this study were 8–10 weeks old and female. All animals were maintained at the Animal Core Facility of Shanghai Jiao Tong University School of Medicine. For the genotyping analysis of mice, the DNA of mouse tissue was extracted and determined by PCR assays with specific primers for Cre and Vps33b^fl/fl^ (Table S1).

### Establishment and analysis of the murine B-ALL model

For establishment of a murine B-ALL model by overexpressing the N-Myc oncogene in hematopoietic stem progenitor cells, an MSCV-N-Myc-IRES-GFP plasmid together with the pCL-ECO packaging plasmid were transfected into HEK293T cells, followed by collection of the supernatant containing retroviruses 48–72 h after transfection. Lin^−^ fetal liver cells were purified and spin infected with N-Myc retroviruses. The infected cells were then incubated in Stemspan medium (StemCell) in the presence of 10 ng/mL murine SCF (Peprotech) and 10 ng/mL murine IL-7 (Peprotech) for 2 days. A total of 1–3 × 10^5^ infected Lin- BM cells were transplanted into lethally irradiated (10 Gy) recipient mice by retro-orbital injection. GFP^+^ BM B-ALL cells collected from primary recipient mice were sorted and transplanted into lethally irradiated 6- to 8-week-old recipients. The frequency of GFP^+^ cells in the peripheral blood of recipient mice was analyzed by flow cytometric analysis at the indicated time points post-transplantation to evaluate B-ALL development. In another set of experiments, 5 × 10^5^ B-ALL cells were cultured in 12-well plates with 500 μL of StemSpan serum-free medium (STEMCELL Technologies) containing 10 ng/mL mouse SCF (Peprotech), 10 ng/mL murine IL-7 (Peprotech), and 30 μg of ANGPTL2-containing SEVs, ANGPTL2-mut#2-containing SEVs or control SEVs for 6 h before transplantation.

### Cell culture and transfection

HEK293T (RRID:CVCL_0063) cells were cultured in DMEM (Thermo Fisher) with 10% FBS (Gibco). In some cases, HEK93T cells were maintained in DMEM with 10% SEV-depleted FBS (Gibco). Cells were plated in antibiotic-free DMEM (high glucose) with 10% FBS before transfection. For generation of ANGPTL2-mCherry-expressing HEK293T cells, a lentiviral plasmid encoding the fusion protein (pLVX-ANGPTL2-mCherry) was transfected into HEK293T cells along with the packaging plasmids pSPAX2 and pMD2.G. The lentiviruses were collected at 48 h and 72 h after transfection, followed by infection with HEK293T cells. For live-cell imaging experiments, pN1-ANGPTL2-mCherry and pN1-CD63-eGFP plasmids were cotransfected into HEK293T cells.

### Flow cytometry

For analysis of N-Myc-induced leukemia development, PB were incubated with anti-CD43-biotin (RRID:AB_466439) followed by with streptavidin-APC (RRID:AB_10366688) and anti-B220-PE (RRID:AB_465671) for 15 min at 4 °C and subjected to flow cytometric analysis. BM cells were incubated with anti-Mac1-PE (RRID:AB_2734869), anti-Gr-1-APC (RRID:AB_469476), anti-B220-PE and anti-CD3e-APC (RRID:AB_469315) for 15 min at 4 °C.

### SEV isolation

For isolation of ANGPTL2-containing SEVs and ANGPTL2-mut#2-containing SEVs, 1.5 × 10^7^ HEK293T cells overexpressing ANGPTL2 or ANGPTL2-mut#2 were cultured in 30 mL of DMEM containing 10% SEV-free FBS for 3 days. The supernatants were collected for further SEV isolation. Sequential centrifugation was performed at 600 × g for 10 min, 2,000 × g for 10 min, and 12,000 × g for 30 min to remove cells and cellular debris. Purified SEVs were washed with serum-free PBS and subsequently pelleted again by ultracentrifugation at 110,000 × g for 80 min. All ultracentrifugation steps were conducted at 4 °C. Purified SEVs were resuspended in 50–100 μL of serum-free PBS, and the related protein concentration was determined using BCA quantification (Thermo Fisher).

### Isolation of SEV proteins and mass spectrometry

Protein extraction and TMT proteomics analysis were supported by HangZhou Jingjie PTM BioLabs. SEV samples were sonicated three times in lysis buffer (8 M urea, 1% Protease Inhibitor Cocktail) on ice using an ultrasonic processor (Scientz). After centrifugation at 18,000 xg, the supernatant was collected, and the protein concentration was determined using a BCA kit. For digestion, the protein solution was treated with 10 mM DTT for 1 h and alkylated with 20 mM IAA for 45 min at room temperature in darkness. After dilution of the urea below 2 M with TMT label buffer, trypsin was added at a 1:50 trypsin-to-protein (m/m) ratio for the first digestion overnight and at a 1:100 trypsin-to-protein (m/m) ratio for the second 4 h digestion. After trypsin digestion, the peptide was desalted on a Strata X C18 SPE column (Phenomenex) and vacuum-dried. The peptides were then TMT labeled according to the manufacturer’s instructions.

The peptides were subjected to NSI source followed by tandem mass spectrometry (MS/MS) in a Q Exactive HF-X (Thermo) coupled with an EASY-nLC 1200 UPLC system. The gradient comprised an increase from 6 to 22% solvent B (0.1% formic acid in 90% acetonitrile) over 47 min, 22% to 32% in 20 min and climbing to 80% in 4 min then holding at 80% for the last 4 min. The electrospray voltage applied was 2.0 kV. The intact peptides were detected in the Orbitrap at a resolution of 120,000. The peptides were then selected for MS/MS with an NCE setting of 28, and the fragments were detected in the Orbitrap at a resolution of 15,000. A data-dependent procedure that alternated between one MS scan followed by 30 MS/MS scans with 30.0 s dynamic exclusion was used. The automatic gain control (AGC) was set at 5E4. The fixed first mass was set as 100 m/z.

The resulting MS/MS data were processed using the MaxQuant search engine (v.1.5.2.8). Tandem mass spectra were searched against the Mus database concatenated with the reverse decoy database. Trypsin/P was specified as a cleavage enzyme allowing up to two missing cleavages. The mass tolerance for the precursor ions was set as 20 ppm in the first search and as 5 ppm in the main search, and the mass tolerance for the fragment ions was set as 0.02 Da. Carbamidomethyl on Cys was specified as a fixed modification. Oxidation on Met and acetylation on the protein N-term were specified as variable modifications. The FDR was adjusted to < 1%, and the minimum score for modified peptides was set as > 40. The minimum peptide length was set at 7. For the quantification method, TMT 6-plex was selected. All the other parameters in MaxQuant were set to the default values.

### Western blot

Cell extracts or SEV extracts were prepared with SDS buffer and were subsequently separated through 10% SDS polyacrylamide gel electrophoresis. The resulting separated proteins were then transferred onto NC membranes (Millipore). The membranes were then blocked with a 5% nonfat milk solution and subjected to incubation with specific primary antibodies, including anti-Flag (Sigma), anti-ANGPTL2 (R&D system), anti-HA (Sigma), anti-VPS33B (Santa Cruz) and anti-Fc (Sigma), followed by incubation with horseradish peroxidase-conjugated secondary antibodies.

### Immunoprecipitation

First, HEK293T cells transfected with the indicated plasmids were subjected to incubation in Pierce immunoprecipitation lysis buffer containing protease and phosphatase inhibitor cocktails (Thermo Scientific) on ice for 30 min. Subsequently, the cell lysates were immunoprecipitated with either anti-HA (Sigma) or anti-Flag M2 beads (Sigma), followed by overnight incubation at 4 °C. For the samples with added anti-HA, Protein A/G agarose beads (30 µL) were added to each sample and incubated for 8 h at 4 °C. The beads were washed with lysis buffer ten times. The immunoprecipitation samples or whole cell lysates were evaluated by Western blot.

### Statistical analysis

The statistical analysis was conducted using version 19.0 of the GraphPad and SPSS software programs. The data are reported as the mean ± standard error (SD). Each experiment was conducted independently no less than three times. Statistical analysis was performed using Student’s t test (two-tailed), one-way analysis of variance (ANOVA) with Tukey's multiple comparison test, or two-way ANOVA with Sidak's multiple comparison test. The level of statistical significance was set at *P* < 0.05 (*P < 0.05, **P < 0.01, and ***P < 0.001).

## Supplementary Information

Below is the link to the electronic supplementary material.

Supplementary file1 (PDF 212 KB)

## Data Availability

All data generated or analysed during this study are included in this published article [19].
