# Supplementary material for: Endothelial-derived small extracellular vesicles support B-cell acute lymphoblastic leukemia development
Source: Cell Oncol (Dordr). 2023 Sep 26;47(1):129–40. doi: 10.1007/s13402-023-00855-0 (PMC10899377; doi:10.1007/s13402-023-00855-0)
Supplement: Supplementary file 1 — Supplementary file1 (PDF 212 KB) [file 13402_2023_855_MOESM1_ESM.pdf]

Supplementary material

Figure S1

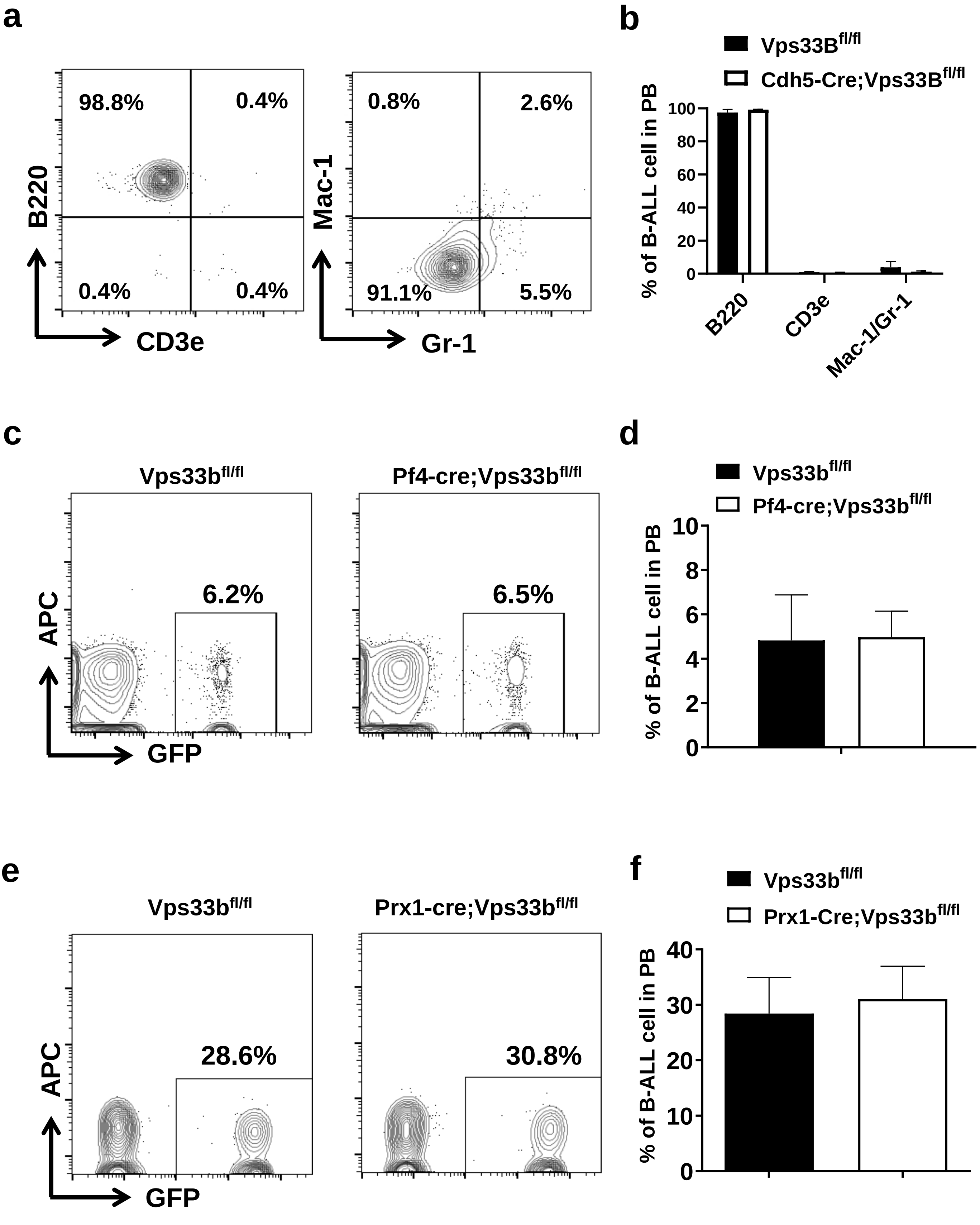

Figure S1. Reduced endothelial cell-derived SEVs secretion prolongs survival in B-ALL mice. (a-b) Representative flow cytometric analysis of the percentages of B220 (B cells), CD3e (T cells) and Mac-1/Gr-1 (myeloid cells) cells in an N-Myc-induced murine B-ALL model (n=3). (c-f) Representative flow cytometric analysis of the percentage of GFP^{+} leukemia cells in the peripheral blood (PB) of recipients (Pf4-Cre;$Vps33b^{fl/fl}$ and Prx1-Cre;$Vps33b^{fl/fl}$ mice) 20 days after transplantation (c, e). Quantification data in Panels c and e are shown (d, f). (n = 4-6; Student's t test).

Figure S2

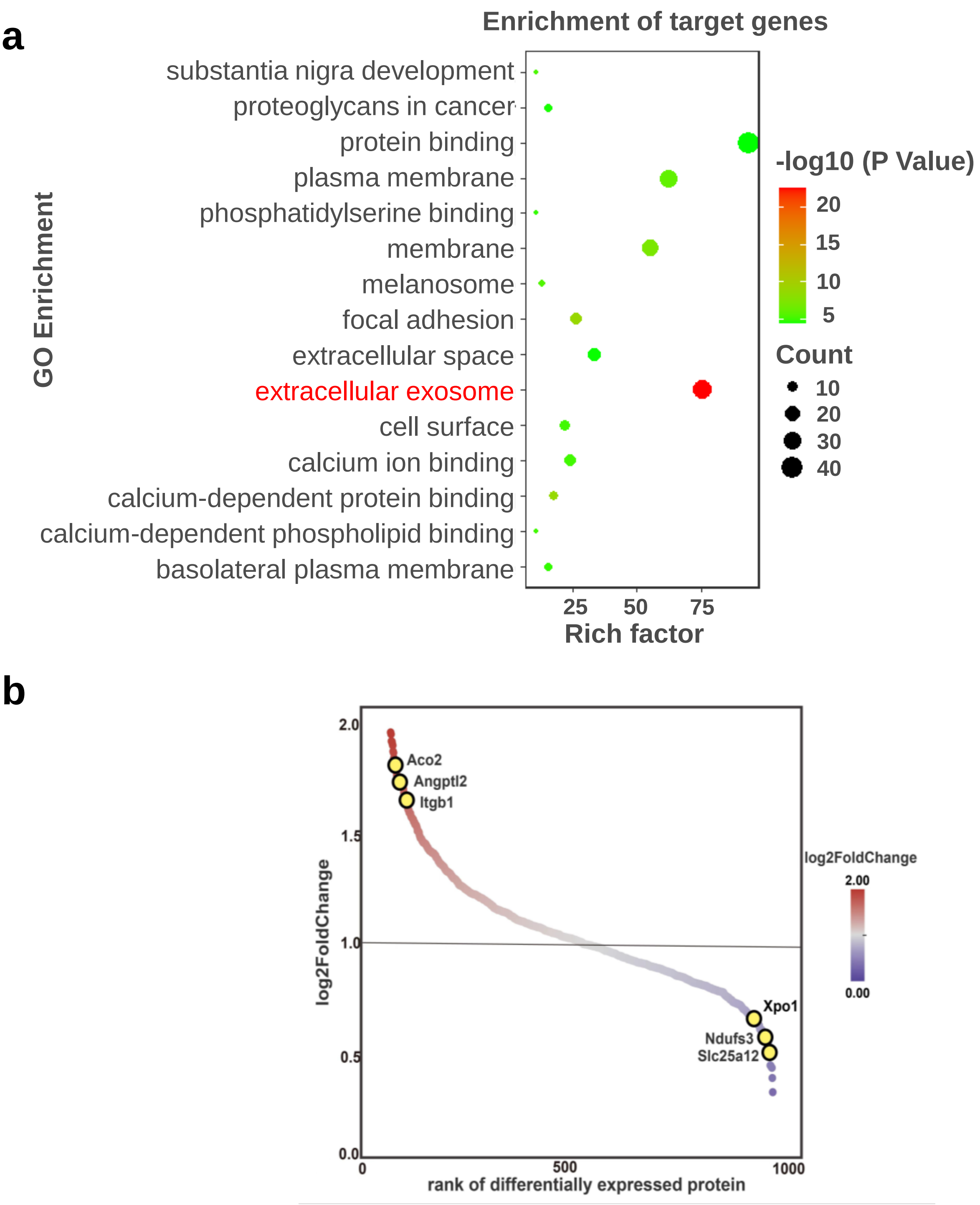
a
Enrichment of target genes
GO Enrichment
substantia nigra development
proteoglycans in cancer
protein binding
plasma membrane
phosphatidylserine binding
membrane
melanosome
focal adhesion
extracellular space
extracellular exosome
cell surface
calcium ion binding
calcium-dependent protein binding
calcium-dependent phospholipid binding
basolateral plasma membrane
25
50
75
Rich factor
-log10 (P Value)
20
15
10
5
Count
10
20
30
40
b
log2FoldChange
2.0
1.5
1.0
0.5
0.0
Aco2
Angptl2
Itgb1
Xpo1
Ndufs3
Slc25a12
0
500
1000
rank of differentially expressed protein
log2FoldChange
2.00
0.00

Figure S2. ANGPTL2-containing SEVs maintain the leukemogenic activities of murine B-ALL cells. (a) GO analyses of the mass spectrometry data of SEVs from WT or Vps33b knockdown murine endothelial cells. Candidate changes are highlighted in red. (b) Analysis of differentially enriched potential proteins in SEVs from WT or Vps33b knockdown murine endothelial cells. Dots in yellow represent significantly changed candidate genes.

Table S1 The primers used in the mouse gene identification

Genotyping Primers	Sequence
Pf4-Cre-F	CCCATACAGCACACCTTTTG
Pf4-Cre-R	TGCACAGTCAGCAGGTT
Cdh5-Cre-F	ATGTCCAATTTACTGACCGTACA
Cdh5-Cre-R	CGCATAACCAGTGAAACAGCATT
Prx1-Cre-F	ATGTCCAATTTACTGACCGTACA
Prx1-Cre-R	ATGTCCAATTTACTGACCGTACA
Vps33b-A1	ATGTCCAATTTACTGACCGTACA
Vps33b-A2	GTATCACTGAGTCACACACATCCA
Vps33b-A3	ATAGAGACGTTAGCAATTCGATCC
Angptl2-loxp-tF	ATCCTAATGTCCCTCTTGGC
Angptl2-FRT-tR	CAGGCTGTGAACAGGTTAGTCATC
Tie2-Cre-F	ATTTGCCTGCATTACCGGTC
Tie2-Cre-R	ATCAACGTTTTCTTTTCGG

Table S2 The primers used in the plasmid construction

Cloning Primers	Sequence
ANGPTL2-C-F	GGAATTCGACAAGCCGTCGGGCCCAT
ANGPTL2-C-R	TGCTCTAGAGTGGAAGGTGTTGGGGTTCG
ANGPTL2-N-F	CCCAAGCTTATGCAGGAGGACGGTTTTGAGGG
ANGPTL2-N-R	GCTCTAGAGGTGGAAGATGGGAGGCTGG
pN1-ANGPTL2-mut-mCherry-F	CCGCTCGAGCAGGAGGACGGTTTTGAGGG
pN1-ANGPTL2-mut-mCherry-R	CCCAAGCTTGTGGAAGGTGTTGGGGTTCG
